# Mining Severe Drug Hypersensitivity Reaction Cases in Pediatric Electronic Health Records: Methodology Development and Applications

**DOI:** 10.2196/37812

**Published:** 2022-09-13

**Authors:** Yuncui Yu, Qiuye Zhao, Wang Cao, Xiaochuan Wang, Yanming Li, Yuefeng Xie, Xiaoling Wang

**Affiliations:** 1 National Center for Children's Health Beijing Children’s Hospital Capital Medical University Beijing China; 2 Bohui Yishu (Beijing) Co, Ltd Beijing China

**Keywords:** drug hypersensitivity reactions, electronic health records, clinical notes, phenotyping, natural language processing, medical language processing, bidirectional encoder representation from transformers

## Abstract

**Background:**

Severe drug hypersensitivity reactions (DHRs) refer to allergic reactions caused by drugs and usually present with severe skin rashes and internal damage as the main symptoms. Reporting of severe DHRs in hospitals now solely occurs through spontaneous reporting systems (SRSs), which clinicians in charge operate. An automatic identification system scrutinizes clinical notes and reports potential severe DHR cases.

**Objective:**

The goal of the research was to develop an automatic identification system for mining severe DHR cases and discover more DHR cases for further study. The proposed method was applied to 9 years of data in pediatrics electronic health records (EHRs) of Beijing Children’s Hospital.

**Methods:**

The phenotyping task was approached as a document classification problem. A DHR dataset containing tagged documents for training was prepared. Each document contains all the clinical notes generated during 1 inpatient visit in this data set. Document-level tags correspond to DHR types and a negative category. Strategies were evaluated for long document classification on the openly available National NLP Clinical Challenges 2016 smoking task. Four strategies were evaluated in this work: document truncation, hierarchy representation, efficient self-attention, and key sentence selection. In-domain and open-domain pretrained embeddings were evaluated on the DHR dataset. An automatic grid search was performed to tune statistical classifiers for the best performance over the transformed data. Inference efficiency and memory requirements of the best performing models were analyzed. The most efficient model for mining DHR cases from millions of documents in the EHR system was run.

**Results:**

For long document classification, key sentence selection with guideline keywords achieved the best performance and was 9 times faster than hierarchy representation models for inference. The best model discovered 1155 DHR cases in Beijing Children’s Hospital EHR system. After double-checking by clinician experts, 357 cases of severe DHRs were finally identified. For the smoking challenge, our model reached the record of state-of-the-art performance (94.1% vs 94.2%).

**Conclusions:**

The proposed method discovered 357 positive DHR cases from a large archive of EHR records, about 90% of which were missed by SRSs. SRSs reported only 36 cases during the same period. The case analysis also found more suspected drugs associated with severe DHRs in pediatrics.

## Introduction

Drug hypersensitivity reactions (DHRs) are one of the adverse drug reactions resembling allergy occurs. DHRs affect more than 7% of the population and are a significant cause of the postmarketing withdrawal of drugs [1]. Severe DHRs, such as anaphylactic shock, drug-induced hypersensitivity syndrome, Stevens-Johnson syndrome, and epidermolysis bullosa, have been observed worldwide with an annual incidence of 0.05 to 3 persons per million population. With mortality rates varying between 5% to 30%, severe DHRs in pediatric populations, including children, infants, and even newborns, comprise 10% to 20% of reported cases [2,3].

Reporting of severe DHRs in hospitals now solely occurs through spontaneous reporting systems (SRSs), which clinicians in charge operate. Previous studies showed that only 10% to 30% of severe adverse drug reactions were reported in SRSs [4]. Even though the missed cases were properly handled and simply not logged into the SRS system, a more thorough report would have helped improve drug guidelines. Recently, routinely collected medical data such as electronic health records (EHRs) are increasingly being used to complement the SRS and enable active pharmacovigilance. EHR systems contain detailed data with timestamps for admissions, discharges, diagnoses, medications, and laboratory tests. However, severe DHR rely on symptoms and signs for detection, which in turn often reside in the free-text areas of EHRs and require the use of natural language processing to extract information.

One of the most well-studied medical language processing applications is phenotyping (eg, the automatic evaluation of phenomics traits such as smoking status) [5]. Automatic identification of severe DHRs in patients can also be explored as a phenotyping task. When no structural data are available, the phenotyping of clinical notes can be formulated as a document classification task, which has been well studied in the natural language processing field.

Recent work [6-8] has reported that clinical documents are too long for contextualized language models to process. Our research group has integrated the medical data from a hospital and established a vertical data warehouse in its early stage. Unlike previous works that only process discharge summaries [5-7], this DHR task deals with documents consisting of all clinical notes associated with 1 inpatient visit. The average word length of discharge summaries is typically hundreds of words. However, in this DHR data set, the average word length is up to several thousand Chinese characters, and some documents contain tens of thousands of Chinese characters. Therefore, picking the best strategy for long document classification is crucial for achieving our objective.

## Methods

### Pipeline Design

This work approaches the automatic identification of DHR cases as a long document classification problem. For training purposes, domain experts prepared a corpus containing document-level tags.

**Figure 1:**
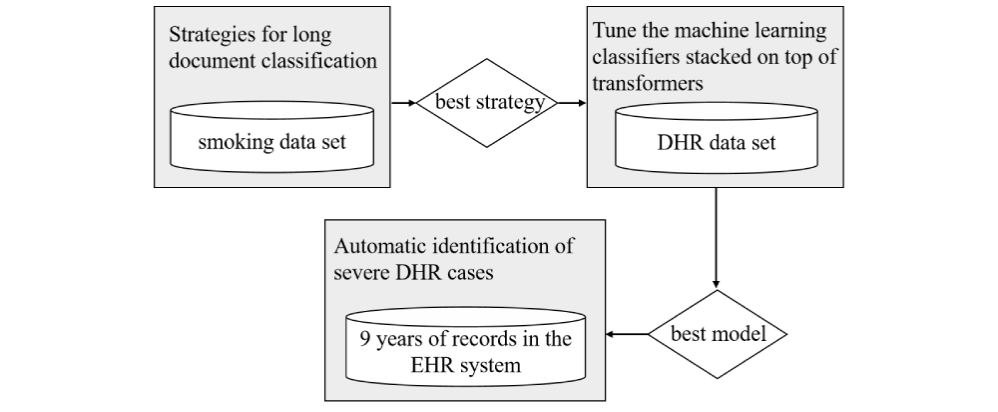
Proposed system pipeline in this study. DHR: drug hypersensitivity reaction; EHR: electronic health record.

Figure 1 demonstrates the proposed system pipeline. First, 4 strategies for long document classification on the openly available smoking task were compared and evaluated. Second, the best strategy for the DHR task was applied. The pretrained embedding models of Chinese medical text on our own DHR task were compared and evaluated. A grid search to tune machine learning classifiers for the best document classification performance on the DHR data set was performed. Finally, the best pipeline to 9 years of data in a paramedic EHR was applied.

### Ethics Approval

The study was reviewed and approved (2019-k-5) by the Institutional Ethics Committee of Beijing Children’s Hospital in China, with a waiver of informed consent.

### Data Set and Metrics

#### Smoking Task

The smoking challenge [5] automatically determines patients’ smoking status from their discharge summaries. The 502 discharge summaries present 5 statuses: past smoker, current smoker, smoker, nonsmoker, and unknown. Following previous work, the class smoker was ignored. Table 1 shows the training and test data distribution.

**Table 1:** The training and test data distribution of the smoking task.

	Past smoker	Current smoker	Nonsmoker	Unknown	Total
Train data set	36	35	66	252	389
Test data set	11	11	16	63	101

#### Severe DHR Task

##### Data Source

Beijing Children’s Hospital’s information system allows for a patient’s history and physician notes to be digitally recorded and instantaneously available via the network to all patient departments. A vertical data warehouse was built based on the integration of medical data in the early stage. It contains 431,972 hospitalization records of 315,608 patients from January 1, 2012, to December 31, 2020, including detailed diagnostic information, medication information, laboratory tests, disease course data, etc. Among them, a hospitalization record represents a hospitalization process. If a patient is hospitalized multiple times, the same patient will have multiple hospitalization records.

##### Corpus Construction

Positive cases that present severe DHRs were collected from 2 pools: the 31 positive cases logged to National Medical Products Administration reporting system and the 183 positive cases discovered by chart review. After deduplication, 200 positive cases were collected. Each positive case was assigned 1 of 4 subcategories. Furthermore, 400 negative cases were randomly sampled from Beijing Children’s Hospital’s EHR system. These cases were assigned a negative (NEG) tag and hand-checked by physicians to ensure they did not present severe DHRs.

The definitions of the 4 subtypes of severe DHR are shown in Multimedia Appendix 1 as found in the Guidelines for Medical Nomenclature Use of Adverse Drug Reactions issued by the Center for Drug Reevaluation of the China National Medical Products Administration in 2016 [9].

##### Training and Test Data Set

These 5 categories of documents were randomly sampled into the training and test data sets. The training and test data distribution is shown in Table 2. The positive and negative ratio is close to the corresponding ratio in the smoking task.

**Table 2:** The training and test data distribution of the severe drug hypersensitivity reaction data set.

	SJS^a^	DIHS^b^	AS^c^	EB^d^	NEG^e^	Total
Training data set	56	44	18	32	323	473
Test data set	18	3	5	7	77	110

^a^SJS: Stevens-Johnson syndrome.

^b^DIHS: drug-induced hypersensitivity syndrome.

^c^AS: anaphylactic shock.

^d^EB: epidermolysis bullosa.

^e^NEG: negative.

#### Evaluation Metrics

The micro-averaged F1 score was used to evaluate the performance of different models following previous study [6]. This metric is used for multiclass classification problems, measuring a balance between precision and recall and giving equal weights to each category.

### Strategies for Long Document Classification

Four strategies were evaluated and compared: document truncation [10], hierarchy representation [6,11], more efficient self-attention [12], and key sentence selection [7,8,13,14]. The best strategy for long document classification was based on the openly available National NLP Clinical Challenges 2016 smoking task results [5]. The results of this task can be more fairly compared to other related works.

#### Document Truncation

The most straightforward way to apply a transformer model with a length limit is to truncate the input and pick the first block of tokens. These models typically require a length limit of 512 words.

#### More Efficient Self-Attention

Self-attention models, such as bidirectional encoder representation from transformer (BERT), require quadratic computational time and space with respect to the input sequence length. The Longformer model uses sparse self-attention instead of full self-attention to process longer documents (up to 4096 tokens).

#### Hierarchy Representation

In a hierarchy approach, sentence representations are built first and then aggregated into a document-level representation. In previous work on the phenotyping task of clinical notes, document representation is built by a sampling layer on top of the BERT blocks of each sentence [6].

#### Key Sentence Selection

A few key sentences could be enough for the document classification task. In previous works, unsupervised methods were explored to generate key sentences, which did not always perform well [13,15]. In this work, the keywords extracted from the task-specific guidelines were explored. The sentences containing keywords were selected as key sentences.

For the smoking task, unigrams and bigrams from previous work were taken as the keyword list: cigarette, smoke, smoked, smoker, smokes, smoking, tobacco [16].

For the DHR task, 2 sets of keywords were evaluated and compared. As an unsupervised method, the term frequency-inverse document frequency (TF-IDF) algorithm computed top feature words. Those containing numbers, foreign alphabets, and special characters were removed from these 2000 words. A total of 163 feature words with a score higher than zero were added to the keyword list.

The parts of the clinical notes that make references to the corresponding guidelines are most relevant for differential classification. Each positive category in the DHR data set is well defined in the corresponding guideline [17-20]. Medical terms were hand-picked from the guidelines. No domain knowledge was required to distinguish medical terms from general text. These keywords are shown in Textbox 1 in Chinese and Textbox 2 in English.

**The guideline keywords for the severe drug hypersensitivity reaction task in Chinese. AS: anaphylactic shock; DIHS: drug-induced hypersensitivity syndrome; EB: epidermolysis bullosa; IVIG: intravenous immunoglobulin; SJS: Stevens-Johnson syndrome; TEN: toxic epidermal necrolysis.**

1. Stevens-Johnson综合征, 过敏性休克, 药物超敏反应综合征, 大疱表皮松解症, AS, EB, TEN, SJS, DIHS
2. 过敏，超敏，黏膜，红斑，松解，喘鸣，支气管痉挛，发绀，呼气流量峰值下降，肌张力减退，荨麻疹，血管性水肿，紫绀，低血容量性低血压，斑疹，斑丘疹，无菌性脓疱，紫癜，剥脱性皮炎，融合成片，松弛性水疱，表皮松解，大疱，表皮剥脱，叶状鳞屑，表皮剥离，猩红热样，麻疹样，弥漫性，黏膜侵蚀，大疱
3. 糖皮质激素，肾上腺素，甲基泼尼松龙，泼尼松，地塞米松， IVIG，甲泼尼龙

**The guideline keywords for the severe drug hypersensitivity reaction task in English. AS: anaphylactic shock; DIHS: drug-induced hypersensitivity syndrome; EB: epidermolysis bullosa; IVIG: intravenous immunoglobulin; SJS: Stevens-Johnson syndrome; TEN: toxic epidermal necrolysis.**

1. Stevens-Johnson syndrome, anaphylactic shock, drug-induced hypersensitivity syndrome, epidermolysis bullosa, AS, EB, TEN, SJS, DIHS
2. Allergy, hypersensitivity, mucous membrane, erythema, epidermolysis, wheezing, bronchospasm, cyanosis, decreased peak expiratory flow, dystonia, urticaria, angioedema, hypovolemic hypotension, macula, maculopapular, sterile pustules, purpura, confluent, flaccid blister, bulla, exfoliative, scales, Scarlet fever–like, measles, diffuse, mucosal erosion, IVIG
3. glucocorticoid, adrenaline, prednisolone, prednisone, dexamethasone, methylpred

#### Data Set With Selected Text

An oracle test was conducted to evaluate whether the strategy of key sentence selection affects performance. This oracle test was performed as follows: (1) for each document that contains any keyword, assign its gold tag, and (2) for all the documents that contain no keywords, assign the UNKNOWN tag (for the smoking task) or the NEG tag (for the DHR task).

As shown in Table 3, key sentence selection reduced the maximum word count and the average word count for both data sets of the smoking task. The oracle micro-F1 was 1.0 for both the training and test set, which meant that the key sentence selection strategy did not affect the overall performance.

Two lists of keywords were evaluated for the DHR task: TF-IDF keywords and guideline keywords. As shown in Table 4, key sentence selection reduced the maximum word count and the average word count for both training and test data sets of the DHR task. The oracle test showed that with TF-IDF keywords, the oracle micro-F1 score was almost 1.0. With guideline keywords, about 2% to 3% of errors in the whole pipeline were introduced by this strategy.

**Table 3:** Statistics on the original and selected text in the smoking task^a^.

		Maximum word count	Average word count	Oracle micro-F1
**Train**				
	Original	3025	766	—^b^
	Selected	194	18	1
**Test**				
	Original	2529	851	—
	Selected	117	18	1

^a^For word counting, all terms split by space delimiters were considered words.

^b^Not applicable.

**Table 4:** Statistics on the original and selected text in the severe drug hypersensitivity reaction task^a^.

Keywords					Maximum average count			Average character count			Oracle micro-F1
**Train**											
	Original			27198			4615			—^b^	
	**Selected**										
		TF-IDF^c^	4681			770			0.99		
		Guideline	1926			199			0.98		
**Test**											
	Original			15454			3963			—	
	**Selected**										
		TF-IDF	3210			687			1		
		Guideline	636			177			0.97		

^a^For the drug hypersensitivity reaction data set, Chinese characters were counted.

^b^Not applicable.

^c^TF-IDF: term frequency-inverse document frequency.

### Transformers

In-domain and open-domain pretrained embeddings by contextualized language models were evaluated in this work. For implementation, the SBERT library [10] computes document embedding with pretrained open-domain or domain-specific language models. There was no fine-tuning conducted for these pretrained models.

This work evaluated the open-domain model bert-base-uncased [21] and domain-specific models ClinicalBERT and DischargeBERT [20] for English clinical notes.

This work evaluated the open-domain model bert-base-chinese [21] and domain-specific model Medbert-kd-chinese [22] for Chinese clinical notes.

### Machine Learning Classifiers

Machine learning classifiers were stacked on top of deep learning transformers. Each machine learning classifier was tuned by 10-fold cross-validation on the training data set. An automatic grid search framework [10] searched for optimal hyperparameters. This work evaluated linear models with stochastic gradient descent (SGD) learning and libsvm for support vector classification (SVC).

## Results

### Smoking Task: Strategies for Long Document Classification

#### Document Truncation

The library SBERT implemented this strategy with pretrained models BERT, ClinicalBERT, and DischargeBERT. As shown in Table 5, these models performed poorly. When long documents were straightforwardly fed into the transformers, only the first 512-word pieces were reserved.

**Table 5:** Phenotyping results (micro-averaged F1) of the smoking task.

Transformer	Classifier	Micro-averaged F1 (%)	
		Original text	Selected text
Longformer	SGD^a^	63.37	78.22
Bert-base-uncased	SGD	67.33	90.01
DischargeBERT	SGD	63.37^b^	91.09
ClinicalBERT	SGD	60.40	94.06

^a^SGD: stochastic gradient descent.

^b^Given the size of the data set, some models may have the same results.

#### More Efficient Self-Attention

The Longformer model uses sparse self-attention instead of full self-attention to process longer documents (up to 4096 tokens). However, as shown in Table 5, it did not outperform BERT baselines.

#### Key Sentence Selection

This work used unigrams and bigrams from Pedersen [16] to select key sentences. As shown in Table 5, each model performs better on the selected text. The domain-specific pretrained language model, ClinicalBERT (91.09%), and DischargeBERT (93.07%) outperformed the open-domain model, bert-base-uncased (90.01%).

#### Hierarchy Representation

In a hierarchy approach, sentence representations are built first and then aggregated into a document-level representation. For a fair comparison, we evaluated and reported the results of previous work [6] with our own evaluation script. As shown in Table 6, the *f*_mean_ architecture in [6] (94.2%) achieved state-of-the-art performance.

As shown in Table 6, our method (94.1%) achieved comparable performance with the top-performing method. Other earlier work for the smoking task (F1 ranged from 77.0% to 90.0%) did not achieve the same level of performance.

The strategies of key sentence selection and hierarchy representation achieve comparable performance. Furthermore, their efficiency and memory requirements were compared. As summarized in Table 7, GPU was not required for training machine learning classifiers in the proposed pipeline. The hierarchy representation model required a Tesla M40 GPU (Nvidia Corp) to train for 1 day. Our method was about 9 times faster than the hierarchy representation model for inference. With the strategies of both documentation truncation and key sentence selection, only 1 block was processed by the transformer models for each document, so the inference time was not reduced by key sentence selection.

**Table 6:** Phenotyping results (micro-averaged F1) of our methods and previous work^a^ of the smoking task.

Transformer	Micro-averaged F1 (%)
ClinicalBERT (ours)	94.1
*f*_mean_ [6]	94.2
Shared task 1st place [23]	90.0
Majority label baseline [6]	81.0
CNN^b^ [24]	77.0

^a^Our method and *f_mean_* were evaluated by the same script over the test data set. Other results were found directly from their published reports. For comparison, the precision of the results is 0.1%.

^b^CNN: convolutional neural networks.

**Table 7:** Runtime and memory requirements of each model. The training time and GPU requirement of *f*_mean_ are taken from previous work [6]. The inference time on the test data set was evaluated on a GPU server with NVIDIA T4 and 4*cpu (Nvidia Corp).

Model	Documents	Inference time on test data set (seconds)	Training time (hours)	GPU memory
*f*_mean_ [6]	text	35.52	24	16
ClinicalBert	text	0.46	—^a^	—
+MLClassifier	selected text	0.437	1	—

^a^Not applicable.

### Severe DHR Task: Stacked Transformers and Classifiers

The smoking task showed that key sentence selection improved self-attention transformers with length limits. In the DHR task, this strategy was evaluated with various transformers and classifiers. As discussed in Methods, 2 kinds of keywords were evaluated and compared. As an unsupervised method, top TF-IDF [8] feature words were used for key sentence selection. Considering that clinical notes comply with guidelines, keywords were drawn from the DHR guidelines.

As shown in Table 8, the guideline keywords always improved the performance, regardless of the stacked transformers and classifiers. The TF-IDF keywords only help with the SVC classifier.

**Table 8:** Phenotyping results (micro-averaged F1) of different transformers for the severe drug hypersensitivity reaction task.

Transformers and classifiers			Micro-averaged F1(%)				
			Original text		Selected text		
					TF-IDF^a^		guidelines
**Bert-base-chinese**							
	SVC^b^	80.91		82.73		87.27	
	SGD^c^	80.00		77.27		86.36	
**Medbert-kd-chinese**							
	SVC	81.82		83.64		89.09	
	SGD	82.73		73.64		87.27	

^a^TF-IDF: term frequency-inverse document frequency.

^b^SVC: support vector classification.

^c^SGD: stochastic gradient descent.

### Applications in a 9-Year EHR

Finally, the best configuration was applied to the 9 years of data in Beijing Children’s Hospital’s EHRs. A total of 1155 cases were alerted. After double-checking by 2 clinicians and 2 pharmacists in pediatrics based on the criterion of severe DHRs, 357 cases of severe DHRs in children were found (Table 9): anaphylactic shock (n=39), drug-induced hypersensitivity syndrome (n=178), Stevens-Johnson syndrome (n=86), and epidermolysis bullosa (n=54). Only 36 of 356 severe DHRs had been reported to SRS before. About 89.89% of cases were underreported, resulting in insufficient attention from drug regulators and clinicians. This suggests that our method could actively identify severe DHRs providing additional evidence for pharmacovigilance in children.

The case analysis indicated many suspected drugs that may cause severe DHRs in pediatrics. The suspected drugs leading to anaphylactic shock mainly included pegaspargase injection, L-asparaginase, cefoperazone sulbactam, etc. Phenobarbital, nimesulide, and cephalosporin antibiotics were the key suspected drugs leading to drug-induced hypersensitivity syndrome and Stevens-Johnson syndrome. In addition, lamotrigine, lysine acetylsalicylate, and meropenem were closely related to the occurrence of epidermolysis bullosa.

**Table 9:** Distribution of the severe drug hypersensitivity reactions cases in 9 years of electronic health records found by the proposed pipeline.

Severe DHR^a^	Reported in SRS^b^ of BCH^c^, n	DHR cases confirmed by experts, (n)		
		Diagnosed in BCH	Diagnosed in other hospitals	Total
AS^d^	4	26	13	39
DIHS^e^	16	29	149	178
SJS^f^	7	9	77	86
EB^g^	9	8	46	54
Total	36	72	285	357

^a^DHR: drug hypersensitivity reaction.

^b^SRS: spontaneous reporting system.

^c^BCH: Beijing Children’s Hospital.

^d^AS: anaphylactic shock.

^e^DIHS: drug-induced hypersensitivity syndrome.

^f^SJS: Stevens-Johnson syndrome.

^g^EB: epidermolysis bullosa.

## Discussion

### Principal Findings

The results showed that clinical documents were too long to perform document classification baselines. Among the 4 strategies of long document classification, hierarchy representation and key sentence selection were best performed on the smoking task. Moreover, key sentence selection was 9 times faster than hierarchy representation models for inference. The keywords extracted from task-specific guidelines performed better than the unsupervised method. Domain-specific language models always performed better than general embeddings.

A total of 1155 cases were alerted, among which clinicians and pharmacists identified 357 cases of severe DHRs in children. Only 36 of these cases have been reported by SRS. This result suggested that the reporting rate of SRS may be as low as 10.08%. The automatic pipeline that scrutinized clinical notes and reported potential severe DHR cases can help decrease the number of missed positive DHR cases and reduce the cost of labor at the same time.

The case analysis also found more suspected drugs associated with severe DHRs in pediatrics. The analysis could help promote postmarketing drug risk assessment conducive to rational drug use and improve drug guidelines.

### Comparison With Prior Work

Our method achieved comparable performance for the smoking task with the top-performing method (94.1% vs 94.2%). For the DHR task, our method discovered 357 positive cases, about 90% of which were missed by SRS.

Recent work has studied that clinical documents are too long for contextualized language models to process [6-8]. Unlike previous works that only process discharge summaries [5-7], this DHR task deals with documents consisting of all clinical notes associated with 1 inpatient visit. The average word length of discharge summaries is typically hundreds of words. However, in the DHR data set, the average word length is up to several thousand Chinese characters, and some documents contain tens of thousands of Chinese characters.

This work has 4 strategies evaluated and compared: document truncation [10], hierarchy representation [6,11], more efficient self-attention [12], and key sentence selection [7,8,13,14]. None of these works considered the use of guidelines.

### Limitations

The proposed method required the annotation of about 200 positive cases for supervised training. When applying to the large archive of EHRs in hospital databases, certain preprocessing steps are still required to prevent malfunctions from badly formatted documents. Such preprocessing steps may vary for each hospital’s system.

### Conclusions

Automatic identification of severe DHRs can be approached as a document classification problem. The best strategy for long document classification of clinical notes is key sentence selection with task-specific guidelines. The reporting of DHR cases cannot only rely on clinicians in charge. In the same period of data, the SRS system reported 36 cases, whereas the automatic process discovered 357 cases. The case analysis also found more suspected drugs associated with severe DHRs in pediatrics.

## Appendix

Multimedia Appendix 1 Types of drug hypersensitivity reactions and criteria.

## Abbreviations

BERT: bidirectional encoder representation from transformer
DHR: drug hypersensitivity reaction
EHR: electronic health record
NEG: negative
SGD: stochastic gradient descent
SRS: spontaneous reporting system
SVC: support vector classification
TF-IDF: term frequency-inverse document frequency
