# Supplementary material for: Mining Severe Drug Hypersensitivity Reaction Cases in Pediatric Electronic Health Records: Methodology Development and Applications
Source: JMIR Med Inform. 2022 Sep 13;10(9):e37812. doi: 10.2196/37812 (PMC9516376; doi:10.2196/37812)
Supplement: Multimedia Appendix 1 [file medinform_v10i9e37812_app1.docx]

**Multimedia Appendix 1. Types of drug hypersensitivity reactions and criteria.**

| Severe DHR | Clinical manifestations | Criterion |
| --- | --- | --- |
| AS | 1.Skin and mucous membrane: transient skin flushing and itching, numbness of lips, tongue and extremities, rash, neurovascular edema, nasal, eye and throat mucous membrane congestion and edema, etc.; 2.Respiratory system: chest tightness, shortness of breath, dyspnea, suffocation, cyanosis, etc.; 3.Cardiovascular system: the blood pressure drops rapidly with the systolic blood pressure below 90mmHg or 20% lower than the basal blood pressure or the pulse pressure difference less than 20mmHg. The patients may also experience palpitations, sweating, and pallor, which may develop cold limbs, cyanosis, weak pulse, tachycardia, and syncope; 4.Nervous system: dizziness, fatigue, vertigo, apathy or restlessness, incontinence, convulsions, coma, etc.; 5.Digestive system: nausea, vomiting, abdominal pain, abdominal distension, diarrhea or severe bloody diarrhea. | At least meet the item 3, accompanied by 1-2 other items; or meet the item 3 with anti-allergic treatment. |
| DIHS | 1.The maculopapular rash that appears more than 3 weeks after medication; 2.After stopping the causative drug, the symptoms persist for more than 2 weeks after drug withdrawal; 3.The body temperature is always higher than 38℃. 4.Associated with liver function damage. (Glutamate aminotransferase>100U/L); 5.Accompanied by one or more of the following hematological changes: ① increased white blood cells (>11×10^9^/L); ② abnormal lymphocytes (more than 5%); ③ increased eosinophils (>1500×10^6^) /L); 6.enlarged lymph nodes; 7.HHV-6 reactivation. | At least meet the item 1 to 5, and the item 4 can also be manifested as severe damage to other organs. |
| SJS | 1.Flu-like prodromal symptoms; 2.Erythema, blisters, bullae and erosions occur on the trunk and limbs; 3.Accompanied by high fever and systemic poisoning symptoms (such as fatigue, increased white blood cells, joint and muscle pain, etc.), as well as liver and kidney and other visceral damage; 4.Erosion of mucous membranes of eyes, nose, mouth and external genitalia; 5.Damage of respiratory tract, digestive tract, kidney and other internal organs. | At least meet the item 2 to 4. |
| EB | 1.The onset of disease is urgent with the rash appearing within 1-4 days and spreading throughout the body rapidly;  2.The rash began with diffuse bright red or purplish red patches, following by flaccid bullae with positive Nikolsky sign; 3.Fever at 39-40℃, accompanied by the damage of liver, kidney, heart, brain, gastrointestinal and other internal organs; 4.Poor prognosis and high mortality. | At least meet the item 1 to 3. |

DHR: Drug hypersensitivity reactions; AS: anaphylactic shock; DIHS: drug-induced hypersensitivity syndrome; SJS: Stevens-Johnson syndrome; EB: epidermolysis bullosa.
